# Case Report: Case of Wernicke’s encephalopathy complicated by postoperative gastroparesis following sigmoid colon malignant tumor surgery with a literature review

**DOI:** 10.3389/fonc.2026.1715017

**Published:** 2026-01-19

**Authors:** Zhengpeng Qian, Dewang Wu, Chengzhang Zhu, Shiyun Xu, Binbin Du, JingJing Li

**Affiliations:** 1 Department of Anorectal Surgery, Gansu Provincial People’s Hospital, Gansu Provincial Clinical Research Center for Anorectal Diseases, Lanzhou, Gansu, China; 2 First Clinical Medical College, Gansu University of Traditional Chinese Medicine, Lanzhou, Gansu, China; 3 First Clinical Medical College, Lanzhou University, Lanzhou, Gansu, China

**Keywords:** gastroparesis, postoperative complications, sigmoid colon malignant tumor, vitamin B1, Wernicke’s encephalopathy

## Abstract

**Objective:**

To explore the pathogenesis, clinical characteristics, and key points of the diagnosis and treatment of postoperative gastroparesis (POG) complicated by Wernicke’s encephalopathy (WE) after sigmoid colon malignant tumor resection so as to improve the understanding of Wernicke’s encephalopathy occurring after gastrointestinal surgery.

**Methods:**

The clinical data of a patient who developed WE due to POG and prolonged fasting following sigmoid colon cancer resection were retrospectively reviewed, and a comprehensive analysis was conducted in combination with relevant literature.

**Results:**

The research team retrospectively analyzed a case of intestinal surgery complicated by gastroparesis and long-term fasting, with the patient subsequently presenting with neurological symptoms such as confusion and somnolence. Initially misdiagnosed as an energy deficiency or intracranial lesions, the diagnosis was confirmed by magnetic resonance imaging (MRI) and therapeutic response.

**Conclusion:**

Gastrointestinal dysfunction and poor nutritional absorption after sigmoid colon resection can induce Wernicke’s encephalopathy. Early clinical identification of this disease is particularly important. Postoperative nutritional management and monitoring of neurological symptoms should be emphasized to enable timely intervention.

## Introduction

Wernicke’s encephalopathy (WE) is an acute neurological disorder caused by thiamine (vitamin B1) deficiency. The classic clinical triad of WE includes altered mental status, ataxia, and ophthalmoplegia. However, only 16%–33% of patients present with this classic triad during initial examination (1). The primary cause of thiamine deficiency is malnutrition, most commonly observed in patients with chronic alcoholism, although a small number of non-alcoholic patients may also be affected (1, 2). In non-alcoholic WE patients, fasting is a common trigger (2). Additionally, conditions such as gastrointestinal surgery, treatment for malignant tumors, and hyperemesis gravidarum may also lead to thiamine deficiency, thereby inducing WE (3–5). There are also reports of WE triggered by electrolyte imbalance and nutritional deficiencies secondary to hyperemesis gravidarum (6, 7). Magnetic resonance imaging (MRI) serves as an important reference for the diagnosis of WE, with typical imaging findings including symmetric T2-weighted hyperintensity in the periaqueductal gray matter of the midbrain, thalamus, and mammillary bodies (2, 7). However, atypical imaging manifestations may also occur, such as cortical damage, which is relatively rare in WE (8). In some cases, patients may exhibit non-typical neurological symptoms, including epileptic seizures, vision loss, or aggressive behavior (9, 10). Timely thiamine supplementation is crucial for improving patient prognosis. Studies have shown that early high-dose thiamine treatment can significantly alleviate core symptoms and may even completely resolve WE manifestations (4). Therefore, early recognition and treatment of WE are critical to prevent permanent neurological damage (11).

## Case report

**Figure 1:**
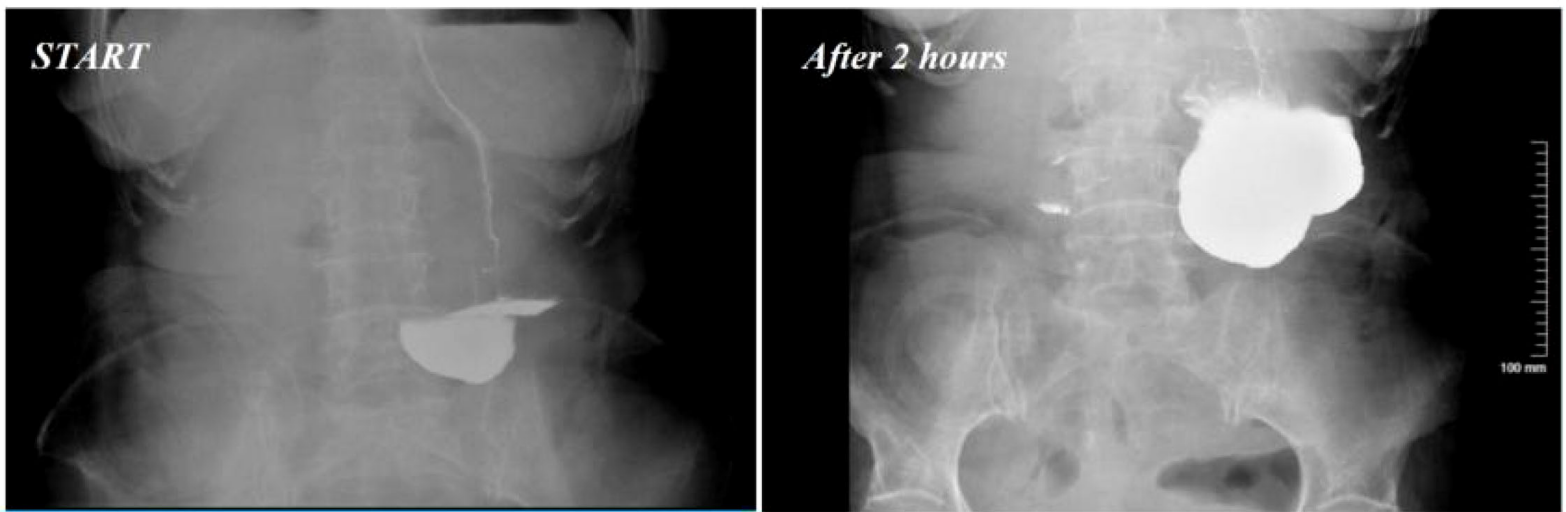
Gastrointestinal angiography on postoperative day 9.

A 77-year-old woman with a Body Mass Index (BMI) of 18.6 kg/m^2^ was admitted to the emergency department on February 19, 2025, due to “cessation of flatus and defecation with abdominal distension for 2 days”, diagnosed with “mechanical intestinal obstruction”. Abdominal computed tomography (CT) revealed diffuse dilatation and fluid accumulation in the small intestine and colon, multiple air-fluid levels, segmental thickening of the bowel wall, and luminal stenosis in the sigmoid colon and proximal rectum, consistent with neoplastic lesions. Her past medical history, obstetric history, and family history were unremarkable, with no history of alcohol abuse or neurological diseases. On physical examination, her blood pressure was 92/72 mmHg; the abdomen was distended with mild tenderness over the entire abdomen, no rebound tenderness, or muscle tension. Digital rectal examination (6-cm insertion) did not palpate a mass, and the glove was stained with blood upon withdrawal. Laboratory tests showed moderate anemia (hemoglobin 86 g/L), thrombocytosis (platelets 398 × 10^9^/L), hypoproteinemia (total protein 63.44 g/L and albumin 36.87 g/L), elevated carcinoembryonic antigen (CEA; 50.39 U/mL), and CA242 (80.91 U/mL). Abdominal CT confirmed intestinal obstruction and a space-occupying lesion in the sigmoid colon; electrocardiogram (ECG) and chest CT showed no abnormalities. Emergency surgery was performed on the same day, including subtotal colectomy, total hysterectomy, left adnexectomy, and ileostomy. Intraoperatively, the sigmoid colon tumor was found to invade the full thickness of the bowel wall and involve surrounding tissues. Postoperative pathology confirmed well-to-moderately differentiated adenocarcinoma of the sigmoid colon (ulcerative type), with cancerous tissue penetrating the serosal layer, metastasis in the pericolonic lymph nodes, and cancerous involvement on the serosal surface of the uterus and cervix, as well as the left ovary. American Joint Committee on Cancer (AJCC) staging was pT4bN1M1. After surgery, the patient was transferred to the intensive care unit (ICU) due to hemodynamic instability and hypoxemia, and her clinical condition stabilized after 7 days of anti-shock treatment and mechanical ventilation support. On postoperative day 9, a sudden onset of nausea and vomiting occurred when she attempted a liquid diet. After conservative treatment failed, postoperative gastroparesis was confirmed by total gastrointestinal angiography (**Figure 1**). Intravenous nutrition was initiated, and a jejunostomy tube was placed for enteral nutrition; however, the patient exhibited poor enteral nutrition tolerance, with persistent nausea and vomiting until postoperative day 25. During this period, her hemoglobin fluctuated between 70 and 86 g/L, and serum albumin remained at 32–37 g/L, suggesting ineffective nutritional support under prolonged fasting.

**Figure 2:**
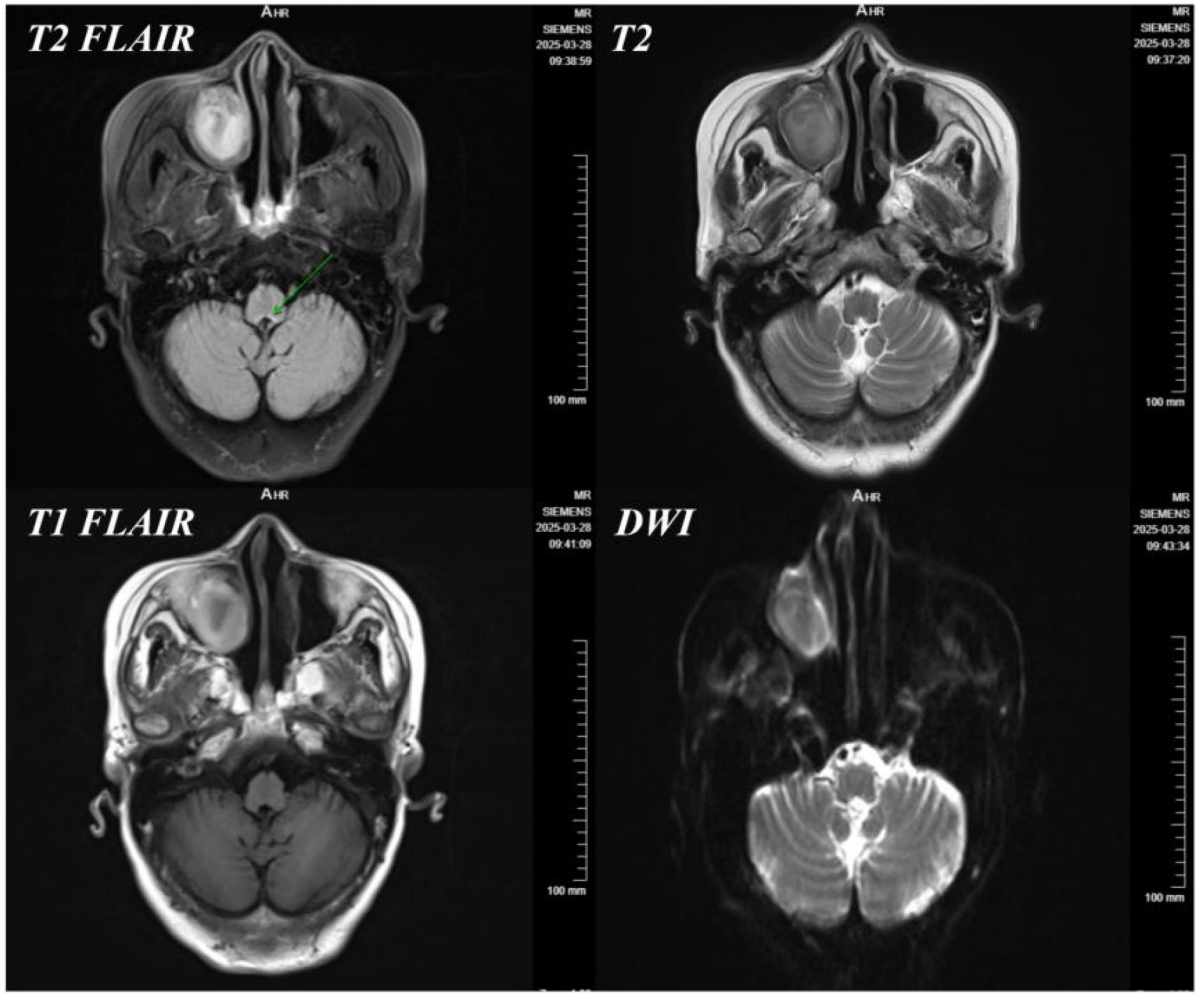
Cranial MRI of the patient on March 28, 2025.

On postoperative day 28, the patient suddenly developed confusion, lethargy, fever (38.5 °C), and diminished pupillary light reflex (other ocular signs could not be evaluated due to poor patient cooperation), accompanied by a sudden decrease in muscle strength to grade 2 in both lower extremities (grade 3 in the upper extremities), with negative pathological signs. Initially, non-specific cerebral dysfunction was considered based on the following factors:

a. Energy metabolism imbalance: Prolonged fasting (19 days postoperatively), hypoproteinemia (albumin 32 g/L), and moderate anemia led to the hypothesis that “insufficient cerebral energy supply resulted in neural inhibition”.

b. Organic intracranial lesions: Cranial MRI revealed “symmetric T2/Fluid-attenuated Inversion Recovery (FLAIR) hyperintensity around the fourth ventricle and bilateral thalami” (**Figure 2**), which indicated metabolic encephalopathy rather than hemorrhage or infarction, although “cerebral metastasis” or “infectious encephalopathy” were briefly considered clinically.

c. Interference from infectious factors: Fever (38.5 °C) with mild elevation of blood count (white blood cell count 11.2 × 10^9^/L) required differentiation between abdominal infection and central nervous system infection, diverting the focus of diagnosis.

As the patient’s condition progressed, the diagnostic and treatment team identified the following key observations: gastroparesis-induced prolonged fasting met the high-risk criteria for WE; somnolence, confusion, and diminished pupillary light reflex were considered manifestations of metabolic encephalopathy. According to Caine’s criteria (12) [a clinical diagnosis of WE can be considered if at least two of the following four symptoms are present: a) nutritional deficiency, b) oculomotor abnormalities, c) cerebellar dysfunction, and d) altered mental status or mild memory impairment], the patient was highly suspected of having WE. Meanwhile, the differentiation between decreased muscle strength and prolonged bed rest or nutritional depletion required correlation with dynamic changes in neurological signs. The symmetric mild lesions around the fourth ventricle observed on MRI highly corresponded to the characteristic brain damage sites of WE (mammillary bodies, periaqueductal gray matter of the midbrain, and thalamus), with metastatic tumor ruled out. Based on these findings, vitamin B1 was administered empirically (100 mg intravenous infusion twice daily). After 3 days of treatment, the patient’s mental state improved significantly (lethargy diminished, and she could follow commands), with restoration of ocular motility. This confirmed the diagnosis of thiamine deficiency encephalopathy, and WE was ultimately confirmed by the therapeutic response. One week after treatment, the patient regained full consciousness, was able to respond to simple questions, and had free ocular movement. Muscle strength in both upper extremities recovered to grade 4, although muscle strength in both lower extremities remained at grade 3 (attributed to concurrent neuromuscular disuse atrophy). Gastroparesis resolved 46 days postoperatively. Follow-up at 1 month postoperatively showed normal cognitive function, no lethargy or cognitive abnormalities, and improved lower extremity muscle strength to grade 4. The patient continued oral thiamine supplementation at 100 mg daily until full independent oral intake was achieved, with no adverse events reported at the 3-month follow-up, indicating that early intervention effectively prevented irreversible neurological damage from WE.

## Discussion

WE is an acute central nervous system metabolic disorder caused by thiamine (vitamin B1) deficiency. Its primary pathological mechanism involves thiamine deficiency-induced impairment of the tricarboxylic acid (TCA) cycle and insufficient ATP production, leading to the disruption of energy metabolism in neurons, lactic acid accumulation, and blood–brain barrier (BBB) disruption, which in turn results in cerebral edema and neuronal damage. Clinically, WE is categorized into alcoholic and non-alcoholic types; the latter is commonly observed in individuals with prolonged fasting, gastrointestinal dysfunction, malnutrition, and other related conditions.

The essence of WE lies in abnormal energy metabolism in the central nervous system triggered by the failure of thiamine-dependent enzyme activity. After being converted into thiamine pyrophosphate (TPP) *in vivo*, thiamine acts as a critical coenzyme for the pyruvate dehydrogenase complex (PDHc) and α-ketoglutarate dehydrogenase complex (α-KGDHc), directly determining the efficiency of the TCA cycle: decreased PDHc activity leads to pyruvate accumulation and its conversion to lactate; reduced α-KGDHc activity further slows the TCA cycle and decreases ATP production (13–15). This energy deficit may further trigger downstream toxic mechanisms: mitochondrial electron leakage causes a reactive oxygen species (ROS) burst and increased superoxide anion production, which attack neuronal membrane lipids and BBB tight junction proteins (e.g., claudin-5), thereby increasing BBB permeability (16–18). The symmetric T2/FLAIR hyperintensities around the fourth ventricle and thalami observed on MRI are typical of WE, involving regions with high dependence on the TCA cycle. These lesions primarily affect the medial vestibular nuclei (leading to nystagmus and vestibular ataxia), thalamus (memory impairment), and reticular formation (encephalopathy)—not limb weakness. Notably, nystagmus is the most common prodromal symptom of WE (more frequent than ophthalmoplegia), often preceding encephalopathy and MRI abnormalities (19, 20). Thiamine (vitamin B1) deficiency in this patient was attributed to the complete cessation of oral intake 27 days postoperatively, exceeding the critical depletion period of endogenous reserves (18–21 days), compounded by inadequate perioperative parenteral thiamine supplementation, which exacerbated the deficiency (17, 21). Notably, thiamine deficiency may also exert a bidirectional effect by contributing to or exacerbating postoperative gastroparesis (POG) through autonomic and neuromuscular mechanisms. As a critical cofactor for cholinergic neurotransmission in the autonomic nervous system that regulates gastrointestinal motility (17), thiamine depletion can impair the coordination of gastric smooth muscle contraction and weaken neuromuscular junction integrity. In this patient with preexisting borderline nutritional reserve (BMI 18.6 kg/m^2^) and malignancy-related catabolism, POG initially restricted oral intake, leading to thiamine deficiency, while subsequent thiamine insufficiency may have further disrupted autonomic regulation of gastric motility, forming a vicious cycle that prolonged POG duration. This mutual interaction is consistent with evidence that nutritional deficiencies, including thiamine depletion, can modulate gastrointestinal function in surgical populations (3, 18). This case can thus be classified as surgical Wernicke’s encephalopathy (SWE), in contrast to alcohol-related Wernicke’s encephalopathy (AWE). The core distinctions between SWE and AWE are as follows:

a. Etiopathogenesis: SWE primarily arises from acute postoperative thiamine depletion, commonly observed following gastrointestinal surgeries (e.g., gastrectomy and bariatric surgery), prolonged fasting, or total parenteral nutrition (TPN) without thiamine supplementation (22), which is consistent with the present case (related to gastrointestinal surgery). Additionally, a subset of non-gastrointestinal-related predisposing factors (e.g., anorexia nervosa, neuromyelitis optica, brain tumors, other types of malignancies, and pregnancy) can also serve as extended etiologies for SWE. In contrast, AWE stems from chronic alcohol-induced damage, where alcohol inhibits intestinal thiamine transporters (THTR-2) and impairs hepatic thiamine storage function, leading to thiamine deficiency (23).

b. Clinical manifestations: Only ≤20% of SWE patients present with the classic triad (ophthalmoplegia, ataxia, and altered mental status); 82% manifest with altered mental status (delirium/coma) as the initial symptom, frequently misdiagnosed as postoperative complications. In AWE, approximately 50% exhibit a relatively typical triad, with ophthalmoplegia being more common (24).

c. Diagnosis and risk of missed diagnosis: SWE has a high missed diagnosis rate (up to 80%, confirmed by autopsy) due to symptoms often attributed to infection, stroke, or anesthesia reactions. A recent case report by Koca et al. (2022) also highlighted the underdiagnosis of non-alcoholic WE in cancer patients, particularly those undergoing gastrointestinal malignancy surgery, which supports the clinical significance of our findings (25). AWE, with its alcohol-related clinical context, garners higher clinical vigilance, reducing the risk of missed diagnosis (26). Both SWE and AWE may show symmetric T2/FLAIR hyperintensity in the medial thalamus and mammillary bodies on MRI; however, chronic AWE may be associated with brain atrophy (27).

d. Prevention and management: SWE requires mandatory perioperative prophylaxis, particularly intravenous thiamine supplementation before gastrointestinal surgery (28). AWE management necessitates strict abstinence from alcohol combined with high-dose vitamin B1 (500 mg TID intravenously); however, patient prognosis may still be impacted by alcohol-related chronic brain injury (e.g., atrophy and cognitive dysfunction) (29).

SWE is an iatrogenic emergency, with prevention as the priority; AWE requires long-term management of alcohol dependence. Both necessitate urgent thiamine therapy, but the subtlety of SWE demands proactive intervention by surgeons for high-risk patients. In this case, the patient presented with acute confusion and symmetric muscle weakness, distinguishing it from the classic WE triad (altered mental status, ophthalmoplegia, and ataxia) (23). Notably, the sudden reduction in lower limb muscle strength to grade 2 could easily be attributed to disuse atrophy from prolonged bed rest, but the MRI findings of brainstem involvement suggested potential neurological compromise.

Differential diagnosis:

a. Energy metabolism imbalance hypothesis: Moderate anemia [hemoglobin (Hb) 86 g/L] and hypoproteinemia [albumin (Alb) 32 g/L] aligned with the “insufficient cerebral energy supply” theory; however, symptom resolution did not occur after correcting anemia and nutritional support, ruling out this diagnosis.

b. Interference from organic intracranial lesions: Despite no hemorrhage or infarction on cranial MRI, clinical suspicion of brain metastasis persisted due to the advanced tumor stage (pT4bN1M1).

c. Misleading infection factors: Fever (38.5 °C) and leukocytosis (11.2 × 10^9^/L) shifted diagnostic focus toward intra-abdominal infection (e.g., anastomotic leak) or central nervous system infection, potentially overlooking the masked thiamine deficiency caused by gastroparesis-induced enteral nutrition intolerance.

After precise vitamin B1 administration and optimized nutritional support, the patient’s mental state fully recovered within 1 week of treatment. However, lower limb muscle strength recovery was delayed (improved from grade 2 to grade 4 at 1 month postoperatively), possibly related to 1) a longer repair cycle for brainstem reticular formation injury (4–6 weeks) and 2) concurrent disuse atrophy of the quadriceps femoris due to reduced isometric contraction. This case highlights insufficient clinical awareness of latent thiamine deficiency in surgical practice, prompting this report to alert peers to remain vigilant about rare complications alongside managing primary surgical conditions.

## Conclusion

This study reports a rare case of Wernicke’s encephalopathy complicating postoperative gastroparesis following sigmoid colon malignancy resection. Prolonged fasting due to postoperative gastroparesis led to thiamine deficiency, manifesting as neurological symptoms initially misdiagnosed but later confirmed by MRI and therapeutic response to thiamine. This underscores that gastrointestinal dysfunction and malabsorption following sigmoid colectomy are significant contributors to WE, emphasizing the importance of early recognition postoperatively. Thus, postoperative care should prioritize nutritional management and neurological symptom monitoring, with timely thiamine supplementation to reduce iatrogenic nutritional deficiencies and prevent irreversible neurological damage.

## Data Availability

The original contributions presented in the study are included in the article/supplementary material. Further inquiries can be directed to the corresponding author.
